# Study design: Pathways to Independence – A study of unaccompanied minor refugees settled in a Norwegian city municipality

**DOI:** 10.1177/14034948211025446

**Published:** 2021-07-02

**Authors:** Ingrid Kvestad, Sølve Bjørn Randal, Nawar Sayyad, Stine Lehmann, Tormod Bøe

**Affiliations:** 1 Regional Centre for Child Mental Health and Welfare, NORCE Norwegian Research Centre, Norway; 2 Child Welfare Services for Unaccompanied Refugee Minors, Bergen Municipality, Norway; 3 Department of Health Promotion and Development, University of Bergen, Norway; 4 Department of Psychosocial Science, University of Bergen, Norway

**Keywords:** Unaccompanied refugee minors, settlement, mental health, survey, child welfare services

## Abstract

**Aims:**

The aim of the ‘Pathways to Independence’ study was to gain knowledge of how to facilitate a healthy development for unaccompanied refugee minors (URMs) after settling in Norwegian municipalities.

**Methods:**

The project is located in the URM child welfare services (URM CWS) of the Bergen municipality. We invited 101 URMs older than 15 years connected to the URM CWS to participate in a comprehensive survey. Of the invited, 81 consented to participate. The survey included questions on the user’s experiences and satisfaction with the URM CWS, and questions related to schooling, social support and activities after settlement. We also included standardized and validated questionnaires on potential traumatic events, mental and somatic health, protective factors and quality of life. These questionnaires have previously been used in two Norwegian epidemiological studies, the ‘Youth@Hordaland’ and ‘Young in Foster care’, facilitating comparison of the results with other youth populations in Norway.

**Conclusion:**

Results from the project will be valuable in the process of reaching knowledge-based recommendations for successful settlement of URMs.

## Rationale for the study

The large number of refugees seeking asylum in Norway in 2015 led to a mobilization of the Norwegian municipalities to increase the rate of settlements. A large portion of these refugees was unaccompanied minors, with 5480 applying for asylum in Norway in 2015 [1]. Many Norwegian municipalities had no previous experience with settlements of unaccompanied refugee minors (URMs), and reports have pointed to the fact that there has been a lack of basic knowledge in the municipalities on factors critical for successful settlement of these minors [2,3].

URM refers to children younger than 18 years at arrival that have fled their country of origin without their parents or other legal guardians to seek protection and asylum in a new country [4]. URMs are characterized by diversity; they differ in terms of ethnicity, in parental and family origin, in their educational level and experience with schooling, and in their reasons for leaving their country of origin [5,6]. Eight out of 10 unaccompanied minors that arrived in Norway in 2015 were above 15 years, more than 90% were boys and the most frequent countries of origin were Afghanistan, Eritrea, Syria and Somalia [1]. Common for these children and adolescents are still that they are not accompanied by their closest caregivers in a significant period of their life, and that there is an increased risk for these children to encounter potential traumatic events both in their country of origin, during their flight and after arrival in their country of settlement [6,7].

There has been an increasing number of studies into the mental health of URMs settled in high-income countries. Results suggest that the URMs are particularly vulnerable in terms of a high burden of mental health problems such as depression, anxiety and post-traumatic stress, and that these mental health problems persist after settlement [8–11]. A longitudinal follow-up of URMs arriving in Belgium, showed that mental health problems remained elevated 18 months after arrival [12], and a recent study of URMs settled in Norway showed that many still reported clinical levels of mental health problems five years after settlement. In this latter study, approximately 24% scored above cut-off for depression symptoms, 15% over cut-off for anxiety symptoms and 42% for post-traumatic stress symptoms five years after arrival [13]. Females and those exposed to more traumatic events were more vulnerable to mental health problems, while older age was associated with higher stability in the level of mental health symptoms. At the five-year follow up, daily hassles were found to be important predictors for mental health problems. These results underline that URMs are a particularly vulnerable group with needs for qualified support upon arrival and during settlement in their host country.

A recent systematic review finds that supportive living arrangements and less restrictive settings are important factors to promote psychological resilience and improved mental health in URMs after settlement [14]. Moreover, the review highlights the fact that most URMs are not receiving appropriate psychological interventions and that there is a lack of systematic evaluation of treatment efficacy and effectiveness in this group of vulnerable adolescents. Other systematic reviews have identified that although basic needs such as safety, shelter and nutrition in general are met for URMs in their host countries, other essential human needs such as the need for stability, for caring and family-like relationships and to be recognized and heard as a unique person seems to be underprioritized by the systems responsible for care upon settlement [15,16].

In Norway, municipalities differ in how they organize the settlement work with the URMs. Most organize this work within the child welfare services (CWS) but some lay the responsibility at other agencies, such as refugee agencies. It is reasonable to believe that the content and quality of the services will be important determinants of a successful transition for the URMs to an independent life in Norwegian society. Still, many point to the fact that there is a lack of systematic knowledge of how municipalities can contribute to a healthy development for URMs during settlement [2,3]. In the city of Bergen, the municipality has for the last two decades organized their settlement work with the URMs within the CWS (the URM CWS). In this context, we have conducted the ‘Pathways to Independence’ study involving URMs 15 years and older, cared for by the URM CWS in the municipality.

## Aims and objectives

The overall aim of the ‘Pathways to Independence’ study was to gain knowledge on how to facilitate a healthy development for URMs after settling in Norwegian municipalities. We conducted a comprehensive survey in URMs settled in Bergen municipality including questions related to experiences and satisfaction with the URM CWS, schooling, social support, and activities after settlement, in addition to standardized and validated questionnaires on potential traumatic events, mental and somatic health, protective factors and quality of life. Based on the results from the survey and subsequent processes in the URM CWS, the overall purpose was to make knowledge-based adjustments to the services provided to the URMs. The main objective of the current paper was to describe the design and procedures of the survey.

## Design and measurement procedures

### Setting

The ‘Pathways to Independence’ study consist of a comprehensive survey conducted among URMs settled in Bergen municipality and co-ordinated by the URM CWS in the municipality. The city of Bergen is the second largest city in Norway, with close to 300,000 inhabitants. Settlement of refugees has over time been a prioritized area in the municipality, and settlement of URMs has been a part of the municipal CWS for the last 20 years. The URM CWS has the sole responsibility for settlements of URMs in the city of Bergen. Their services include both case work and placements in foster homes, institutions, joint homes, host families and private housing with or without follow up. Each URM has close contact with their case worker at the URM child welfare agency, and services may be offered until the URM turns 25 years.

### Participants

All URMs 15 years or more associated with the URM CWS in Bergen municipality were invited to participate in the study, and we included all who were 15 years or older and consented to participate in the study. For participants younger than 16, consent was also obtained from their legal guardian. From the target population of 116 URMs, 10 were considered ineligible to participate due to evasive behaviour and poor mental health, three were excluded due to inability to provide the participants with proper follow-up after the survey (participants living in other parts of the country), and two were excluded due to lack of capacity of the case worker. Hence, the number of invited URMs was 101 of which 81 consented, yielding a participation rate of 80%. Demographic information of the participants is provided in Table I. More than 80% of the participants were males, the most frequent country of origin was Afghanistan (47%), followed by Eritrea, Syria and Somalia. The mean age of the participants was 18 and the age ranged from 15 to 20 years. The 20 URMs that declined participation in the study, were comparable to the study sample in terms of gender and country of origin (greatest share from Afghanistan, some from Eritrea and a few from ‘Other’). A greater share of URMs that declined were younger and older (15–16 and 19–20 years), while fewer were between 17–18 years than in the study sample. Some of these URMs declined, responding that they did not want to think about difficulties in the past, others did not respond to the invitation or did not collaborate to schedule a time to do the survey, others said ‘no’ without providing a reason.

**Table I.** Demographic information of the unaccompanied refugee minors (*n* = 81).

		SD	Min	Max
**Gender**				
Boys (*n* (%))	67 (82.7)			
**Country of origin (*n* (%))**				
Afghanistan	38 (46.9)			
Syria	8 (9.9)			
Eritrea	14 (17.3)			
Somalia	7 (8.6)			
Other (Ethiopia, Palestine, Kongo, Sudan)	14 (17.3)			
**Age (years)**				
Current age (*n* (%))				
15–16	10 (12.4)			
17–18	38 (46.9)			
19–20	33 (40.7)			
Mean	18	1.3	15	20
Mean age when left country of origin	13.5	3.0	0	17
Mean age at arrival in Norway	14.5	2.6	0	17
Mean age at arrival in Bergen	15.5	2.0	6	17

### Procedure

The enrolment and data collection lasted from December 2018 to January 2019. The case workers at the URM CWS were responsible for inviting the URMs to participate in the study and to schedule a time to complete the comprehensive survey administrated online using Qualtrics Survey Software. The URMs completed the online questionnaire at the case worker’s office. URMs were first provided with written and oral information about the study and consented to participate on the first page of the questionnaire, after going through the information thoroughly with the case workers. The case workers were present and available for questions and queries while the URMs filled in the survey but were instructed to not look at the participants’ responses. Six of the participants used an interpreter due to lack of knowledge of the Norwegian language. Case workers clarified words and sentences that the URMs found difficult to understand if an interpreter was not used. Participants used between 1.5 to 3.5 hours to complete the survey.

### Instruments

The survey was developed particularly for this study and included a total of 213 questions. These questions were related to background information (including demographic information such as age, gender and country of origin, but also current type of housing (i.e. foster care, group homes, institution, host family, living alone) and the number of times the URM had moved after settlement), the URMs’ experience and satisfaction with the URM CWS, their schooling experiences, perceptions of social support and participation in activities after settlement. In addition, we included standardized and validated questionnaires measuring exposure to potential traumatic events, symptoms of mental health problems and somatic complaints, sleep patterns and problems, protective factors, quality of life and acculturation strategies (see Table II for a detailed presentation). Many of these questionnaires have previously been used in the Norwegian epidemiological studies ‘Youth@Hordaland’ (https://www.norceresearch.no/prosjekter/barn-i-bergen) and ‘Young in Foster care’ (https://www.norceresearch.no/prosjekter/fosterbarns-psykiske-helse), thus facilitating comparisons of the URMs’ responses to youth from a general population and youth in foster care in Norway.

**Table II.** Overview of the domains and questionnaires in the ‘Pathways to Independence’ study.

Domain	Description	Number of items	Youth@Hordaland	Young in Foster care
Background	Questions on the unaccompanied refugee minors’ (URMs’) background and demography such as sex, country of origin, age (current, when leaving country of origin, at arrival to Norway) and living arrangements in Bergen.	7		
URM child welfare services (CWS)	We constructed a questionnaire on the experience and satisfaction with the work of the CWS provided to the URMs. We also asked questions on the number of contacts with the case workers during the past month, whether there had been changes in the case worker and how many times the URM had moved after settling in Bergen municipality.	13		
Social support and Schooling activities	We asked questions on perceived social support such as contact with family (in country of origin and in Norway), friends and activities.	7		
	A range of questions on schooling and school satisfaction was made particularly for the current study.	19		
Acculturation strategies	Acculturation strategies involves the way immigrants prefer to relate to the society of settlement (cultural adoption) and their country of origin (cultural maintenance). In the current study, we used the ‘Vancouver Index of Acculturation’ (VIA) to assess the cultural strategies of the URMs. The VIA consists of 20 statements and the respondent rate to what extent they agree or disagree on each statement on a Likert scale. The VIA provides one score on cultural adoption and one on cultural maintenance [18].	20		
Potential traumatic events (PTEs)	We used an adjusted version of the ‘Child and Adolescent Trauma Screen’ (CATS) to assess PTEs and reactions to these [19]. Part 1 assess 15 different forms of PTEs young people may experience both outside of and within a family context, as defined in the *Diagnostic and Statistical Manual of Mental Disorders* (*DSM-5*). In addition, three items based on items 1, 4 and 5 in the Adverse Childhood Experiences (ACE) questionnaire covering emotional neglect, physical neglect and emotional abuse were included [20]. Further, two custom made items assessed parentification due to neglect.	15		X
Mental health	Part 2 of the CATS comprises 20 items covering symptoms of post-traumatic stress disorder (PTSD) and is based on the *DSM-5* criteria. Accordingly, items cover the following core symptoms of PTSD: intrusions, avoidance, negative alterations in cognition and mood, and hyper-arousal. The PTSD part is scored Never (= 0), Once in a while (= 1), Half of the time (= 2) and Almost always (= 3), yielding a sum score ranging from 0 to 60.	20		X
	General mental health was assessed with the ‘Strengths and Difficulties Questionnaire’ (SDQ) [21] which is a screening tool to detect symptoms in four areas of problems (emotional problems, conduct problems, peer problems and attention-deficit hyperactivity) as well as prosocial behaviours. The SDQ consists of 25 items, and each subscale is measured with five items each, scored on a scale with response options ‘Not true’, ‘Somewhat true’ and ‘Certainly true’. In addition to the subscale scores, a total difficulties score is computed by adding the scores from the four problem scales.	30	X	X
	The short version of the ‘Moods and Feelings Questionnaire’ (SMFQ) [22] was used to measure symptoms of depression. The SMFQ consists of 13 statements (e.g. ‘I am feeling low’, ‘No one likes me’, etc.) that the participants respond to using response categories ‘Not true’, ‘Sometimes true’, or ‘True’.	13	X	X
Health complaints	Health complaints were measured using the ‘Health Behavior in School-Aged Children Symptoms Checklist’ (HBSC-SCL) [23]. The participants were asked to report on the frequency of headache, abdominal pain, back pain, dizziness and neck/shoulder pain on a five-point scale ranging from 0, ‘rarely or never’, to 4, ‘about every day’.	5	X	
Sleep	Insomnia was measured by a self-report operationalization of the *DSM-5* insomnia disorder previously used in the same age group [24]. Time in bed for weekdays and weekends, sleep onset latency and wake after sleep onset was assessed and allows for calculations of sleep duration. In addition, perceived sleep need, daytime sleep and oversleeping were assessed by single items.	9	X	X
Protective factors	‘Resilience Scale for Adolescents’ (READ) is a 28-item scale that is originally organized into five subscales: Personal Competence, Social Competence, Structured Style, Family Cohesion and Social Resources [25]. The items are rated on a five-point Likert scale ranging from totally disagree to totally agree. Higher scores indicate a higher level of protection. The items from the Family Cohesion subscale were not included in the current study, and we used a different five-factor model identified in the Youth@Hordaland data including two new personal factors: Goal Orientation and Self-Confidence [26].	28	X	X
Health related quality of life	The ‘KIDSCREEN-27 Quality of Life Questionnaire’ [27] consists of 27 items measuring five areas of quality of life during the last week: physical well-being, psychological well-being, parent relations and autonomy, peers and social support, and school environment. Each item is scored on a five-point Likert scale. The ‘KIDSCREEN-10 Questionnaire’ [28] is embedded within the KIDSCREEN-27 and comprise10 items yielding a singular index of general Quality of Life.	27		X

Ahead of the data collection, we piloted the survey on three young adult URMs that were previous users of the URM CWS. Following this pilot, we adjusted the questionnaire based on the feedback, primarily regarding difficult wording, length of the questionnaire and by omitting topics perceived as irrelevant.

## Ethics and dissemination

The project was approved by the Regional Committee for Medical and Health Research Ethics of Western Norway (REK-Vest) (2018/966) and conducted in accordance with recommendations from the Norwegian Data Protection Services. Participation in the study was voluntarily, and the participants could withdraw from the project at any time. All participants received a gift certificate of 300 NOK (*c.* €30) as a compensation for their participation.

The project group consisted of experienced clinicians and researchers that have followed the procedures closely to ensure that the study was conducted according to high ethical standards. After the URMs had completed the questionnaire, the case workers were instructed to be available for follow-ups when needed. Procedures were made for referrals to further counselling outside of the URM CWS in case of severe emotional reactions.

Selected descriptive results from the study have been disseminated to the Bergen municipality and also to other Norwegian regional and national policy makers in a report [17] and through oral presentations. All participants were also invited for a meeting to get feedback on their results and to discuss the findings. A total of 21 URMs joined this meeting, of whom five were recruited for smaller group discussions on the results and on recommendations for the CWS on necessary adjustments of the services provided. Further results will be published in peer-reviewed journals.

## Statistics

The sample of URMs is limited and therefore somewhat restrictive regarding analytical opportunities. Still, the descriptive results from the survey have already provided the CWS with detailed and actionable knowledge about important areas of concern not previously uncovered systematically. Also, by aligning the design of the study with existing studies in terms of the instruments used, we facilitate matched comparisons with youth from the general population and foster children on important areas such as mental health problems and risk and resilience factors. Group comparisons could, for example, identify particular areas where the needs of URMs depart from other youths, which is essential knowledge when aiming to tailor services for this particular group of youth.

## Potentials

The overall aim of the current project was to gain more knowledge on how to facilitate a healthy development for URMs after settlement in Norwegian municipalities. The project has the potential to reach this aim through two main processes: first through increased knowledge about this group of youth through the dissemination of results in national and international peer reviewed journals, and secondly, through collaborative processes within the URM CWS in the Bergen municipality aiming to improve the quality of services, with transfer value to other municipalities and connected agencies in Norway.

The survey provides a comprehensive data source of the needs and recourses of URMs after settlement in a Norwegian municipality. Moreover, the inclusion of standardized and validated instruments that have been used in previous Norwegian epidemiological studies gives a unique opportunity to provide a comparative perspective on the situation of the group of URMs. Hence, results from the study can be valuable in the process of reaching empirically based recommendations on how to succeed with URMs after settlement in Norway, with the potential to fill in important knowledge gaps [3]. This group of youth are vulnerable, and the burden of mental health problems are known to be high [8,10,12,13]. Improving the settlement process may benefit their health, and thereby also contributing to improved public health in the host countries.

An important part of the current project has been the dissemination of the results from the survey directly to the URM CWS both in oral presentations and through a report [17]. The results were presented and discussed in all parts of the services, including the management team, team of case workers, social workers and to the participants themselves. The aim of the dissemination has been to facilitate discussions on how to meet the needs of the URMs that were identified in the survey within the provided services, a process that is still ongoing.

The close collaboration between the researchers, service providers and users has been essential and present in all parts of the project. Users and service providers were involved in the selection and piloting of the questionnaires, in designing the study procedures, in administering the survey, in the interpretation and dissemination of the results, and they will be central when implementing changes to the services to URMs that have been informed from the knowledge gained in the survey and project. The difference in expertise and knowledge in this group of collaborators has been an important condition for the success of this study.

## Strengths and limitations

A notable strength of the current study is that it contains a comprehensive assessment, using validated instruments, of a population that is hard to reach, and the high participation rate (80%). Still, a notable weakness is the relatively few participants, in particular females, which restricts the potential for doing bi- and multivariate analyses and subgroup analyses to identify specific associations with, for example, country of origin and sex. Moreover, questionnaires were in Norwegian and many of the topics could have a culturally specific character that may be difficult to transfer across cultures. Using common questionnaires with that of other studies in important comparison groups in Norway is a unique asset of this study. It could be argued that the presence of the case workers whilst conducting the study, particularly during the completion of the survey, could bias responses to some questions. Still, through close discussion in the project group and with the ethical board, it was decided that the case workers were important measures to secure a safe situation for the URMs when answering potentially emotionally challenging questions.

## Collaboration

The dataset is administered by Regional Centre for Child Mental Health and Welfare, West (RKBU-Vest), NORCE Norwegian Research Centre. Researchers are invited to contact the project leader (first author) for enquires regarding collaboration. Approval from a Norwegian regional committee for medical and health research ethics and the Norwegian data protection services is a pre-requirement.
